# Interpretive agreement of susceptibility between broth microdilution and disk diffusion methods for cefiderocol, using criteria from the Clinical and Laboratory Standards Institute, European Committee on Antimicrobial Susceptibility Testing, and the Food and Drug Administration

**DOI:** 10.1128/jcm.01255-25

**Published:** 2025-12-10

**Authors:** Yu-Tzu Lin, Hsiu-Hsien Lin, Chih-Hao Chen, Kun-Hao Tseng, Mao-Wang Ho, Po-Ren Hsueh

**Affiliations:** 1 Department of Medical Laboratory Science and Biotechnology, China Medical University 38019, Taichung, Taiwan; 2 Department of Laboratory Medicine, China Medical University Hospital, China Medical University 38020, Taichung, Taiwan; 3 Division of Infectious Diseases, Department of Internal Medicine, China Medical University Hospital, China Medical University 38020, Taichung, Taiwan; Maine Medical Center Department of Medicine, Portland, Maine, USA

**Keywords:** cefiderocol, breakpoints, antimicrobial susceptibility testing, carbapenem-resistant Gram-negative bacilli

## Abstract

Cefiderocol (FDC) is a siderophore cephalosporin approved for the treatment of infections caused by multidrug-resistant Gram-negative bacilli (MDR-GNB), including carbapenem-resistant (CR) isolates. However, interpretation criteria for both broth microdilution (BMD) and disk diffusion (DD) methods differ among Clinical and Laboratory Standards Institute (CLSI), European Committee on Antimicrobial Susceptibility Testing (EUCAST), and U.S. Food and Drug Administration (FDA) guidelines. This study aimed to assess the categorical agreement (CA) between minimum inhibitory concentrations (MICs) determined by the BMD method and inhibition zone diameters measured by the DD method for FDC, using different interpretive breakpoints across various Gram-negative bacilli (GNB) isolates. A total of 1,170 GNB isolates were collected, including CR and carbapenem-susceptible (CS) isolates of *Escherichia coli*, *Klebsiella pneumoniae*, *Klebsiella oxytoca*, *Enterobacter cloacae* complex, *Pseudomonas aeruginosa*, and *Acinetobacter baumannii-calcoaceticus* complex. Isolates of *Stenotrophomonas maltophilia*, *Burkholderia cepacia*, and *Elizabethkingia anophelis*—which are not categorized by carbapenem susceptibility—were also included. The CA and error rates (minor error, major error, and very major error) were calculated using MIC interpretation as the reference. Among CR isolates, CA based on CLSI breakpoints ranged from 90.0% to 100% across species. U.S. FDA criteria showed comparable performance, except for *A. baumannii-calcoaceticus* complex, where CA was lower (63.0%). In contrast, EUCAST breakpoints demonstrated greater variability, with CA ranging from 66.7% to 100%. Among CS isolates, CA was generally high (≥87.5%) across most standards and species, with exceptions including EUCAST in the *E. cloacae* complex (63.0%) and U.S. FDA in *A. baumannii-calcoaceticus* complex (73.7%). In conclusion, CLSI breakpoints demonstrated the highest consistency between DD and BMD methods.

**IMPORTANCE:**

Cefiderocol is one of the few remaining treatment options for infections caused by carbapenem-resistant Gram-negative bacilli (GNB). Accurate interpretation of susceptibility testing is essential for guiding effective therapy. However, discrepancies among breakpoints established by the Clinical and Laboratory Standards Institute, the European Committee on Antimicrobial Susceptibility Testing, and the U.S. Food and Drug Administration may result in inconsistent interpretations, particularly when using the disk diffusion (DD) method, which is more practical in many clinical laboratories. This study compares the categorical agreement between broth microdilution and DD method interpretations for Gram-negative bacilli across multiple guidelines and highlights the potential for misclassification. It also provides susceptibility data for *Burkholderia cepacia* and *Elizabethkingia anophelis*, for which established interpretive criteria are currently lacking.

## INTRODUCTION

Carbapenems are currently the drugs of choice for the treatment of infections caused by multidrug-resistant Gram-negative bacilli (MDR-GNB). However, the widespread use of carbapenems has led to the emergence of carbapenem-resistant (CR) GNB, which have become a serious global public health concern. CR-Enterobacterales and CR-*Acinetobacter baumannii* are classified in the “critical” priority tier of the World Health Organization Bacterial Priority Pathogens List, while CR-*Pseudomonas aeruginosa* is listed in the “high” priority tier (1). In addition, pathogens such as *Stenotrophomonas maltophilia*, *Burkholderia cepacia*, and *Elizabethkingia anophelis*, which are opportunistic agents in immunocompromised patients, present major clinical challenges due to their multiple intrinsic and acquired resistance mechanisms (2–4).

 Cefiderocol (FDC) is a catechol-substituted cephalosporin that combines a cephalosporin core with a siderophore side chain, enabling it to penetrate the bacterial periplasmic space via a “Trojan horse” mechanism (5). Siderophores are iron-chelating compounds secreted by bacteria to facilitate iron uptake, which is essential for their growth (6). FDC binds ferric iron and exploits bacterial siderophore transport systems to gain entry into the cell, thereby achieving high concentrations in the periplasmic space (7, 8). Once inside, it binds with high affinity to penicillin-binding proteins, inhibiting cell wall synthesis and exerting potent antibacterial activity. By utilizing the bacterial iron transport system, FDC can circumvent resistance mechanisms such as porin loss and efflux pump overexpression (9, 10). In addition, its C-7 side chain enhances outer membrane penetration and confers stability against hydrolysis by a wide range of β-lactamases (11–13). FDC exhibits potent activity against strains producing all four Ambler classes of β-lactamases, including carbapenemase. Consequently, it is effective in the treatment of infections caused by MDR-GNB, including MDR-Enterobacterales, *P. aeruginosa*, *A. baumannii*, and *S. maltophilia* (9, 10, 14). It has been approved by the U.S. Food and Drug Administration (FDA) for the treatment of complicated urinary tract infections and hospital-acquired and ventilator-associated bacterial pneumonia (15, 16).

 Because FDC minimum inhibitory concentration (MIC) testing is not yet implemented in most automated susceptibility systems, the disk diffusion (DD) method remains a practical alternative for many clinical laboratories. However, differences in testing methodology (MIC vs DD) and interpretive standards (Clinical and Laboratory Standards Institute [CLSI], European Committee on Antimicrobial Susceptibility Testing [EUCAST], or FDA) across laboratories and regions can lead to variability in susceptibility categorization and hinder direct comparison among studies (17–19). Such methodological and interpretive discrepancies may result in variable clinical interpretations and potential misclassification, ultimately affecting treatment decisions. Furthermore, breakpoints remain unavailable for certain species. Therefore, this study aimed to compare the categorical agreement (CA) between the broth microdilution (BMD) and DD methods results for multiple clinically relevant species with a high need for FDC therapy, according to different interpretive criteria, and to describe the MIC and inhibition zone distributions for species lacking breakpoints, namely, *B. cepacia* and *E. anophelis*.

## MATERIALS AND METHODS

### Bacterial isolates

A total of 1,170 Gram-negative bacilli isolates were included in this study, comprising the following species and numbers: *Escherichia coli* (*n* = 200, including 100 CR and 100 carbapenem-susceptible [CS] isolates), *Klebsiella pneumoniae* (*n* = 300; 200 CR, 100 CS), *Klebsiella oxytoca* (*n* = 40; 20 CR, 20 CS), *P. aeruginosa* (*n* = 100; 60 CR, 40 CS), *Enterobacter cloacae* complex (*n* = 100; 60 CR, 40 CS), *A. baumannii-calcoaceticus* complex (*n* = 300; 200 CR, 100 CS), *S. maltophilia* (*n* = 60), *E. anophelis* (*n* = 50), and *B. cepacia* (*n* = 20). All isolates were randomly collected from patients receiving care at China Medical University Hospital between September 2018 and March 2025. Bacterial identification was conducted using a matrix-assisted laser desorption/ionization time-of-flight mass spectrometry Biotyper System (Bruker Microflex LT/SH; Bruker Daltonics GmbH, Bremen, Germany), and antimicrobial susceptibility testing was performed with the Phoenix Automated System (Becton-Dickinson Microbiology Systems, Sparks, MD, USA) for routine antimicrobial agents, including carbapenems but not FDC. CR isolates were defined as those classified as non-susceptible (intermediate or resistant) to at least one carbapenem, according to the CLSI guidelines (17). For Enterobacterales, CR was defined as ertapenem MIC ≥1 µg/mL, imipenem MIC ≥2 µg/mL, or meropenem MIC ≥2 µg/mL. For *P. aeruginosa* and *A. baumannii-calcoaceticus* complex, CR was defined as imipenem or meropenem MIC ≥4 µg/mL.

### Inhibition zones of FDC

The inhibition zone diameters of FDC were determined using the DD method designed to comply with both CLSI M02 (20) and EUCAST disk diffusion method manual (21) requirements. Bacterial suspensions were adjusted to a 0.5 McFarland standard and inoculated onto Mueller–Hinton agar (BD Difco, Sparks, MD, USA), which are not iron-depleted, in accordance with CLSI (20) and EUCAST (21, 22) recommendations for FDC testing. A 30 µg BD BBL Sensi-Disc cefiderocol disk (Becton Dickinson, Sparks, MD, USA) was placed on each plate. The plates were incubated at 35°C for 18 h for *E. coli*, *K. pneumoniae*, *K. oxytoca*, *E. cloacae* complex, and *P. aeruginosa*, and for 20 h for *A. baumannii-calcoaceticus* complex, *B. cepacia*, and *S. maltophilia*. For *E. anophelis*, for which neither guideline specifies an incubation duration, an 18 h incubation was used based on previous reports evaluating *E. anophelis* susceptibility testing (23). Zone diameters were interpreted according to both the CLSI M02 (20) and the EUCAST Reading Guide for Disk Diffusion Method (version 11.0) (24), which provide identical criteria for resolving double or fuzzy inhibition zones. Quality control was confirmed using *E. coli* ATCC 25922 and *P. aeruginosa* ATCC 27853. For ATCC 25922, QC results ranged from 25 to 30 mm (acceptable ranges: CLSI 25–31 mm, EUCAST 24–30 mm); for ATCC 27853, QC results ranged from 22 to 29 mm (acceptable ranges: CLSI 22–31 mm, EUCAST 23–29 mm) (17, 25).

### MICs of FDC

The MICs of FDC were determined using the BMD method with the Shionogi MIC Dry Plate Cefiderocol (Shionogi & Co., Ltd., Osaka, Japan), following procedures consistent with both CLSI M07 (26) and the EUCAST Guidance document on broth microdilution testing of cefiderocol (22). Twofold serial dilutions of FDC (final concentrations 32–0.03 µg/mL) were prepared in iron-depleted cation-adjusted Mueller–Hinton broth. A bacterial suspension was prepared by diluting 0.05 mL of a standardized inoculum into 12 mL of sterile distilled water, and 0.1 mL of the diluted suspension was added to each well. The plates were incubated at 35°C for 18 h for *E. coli*, *K. pneumoniae*, *K. oxytoca*, *E. cloacae* complex, *P. aeruginosa,* and *E. anophelis*, or 20 h for *A. baumannii-calcoaceticus* complex, *B. cepacia*, and *S. maltophilia*, matching the conditions used for disk diffusion testing. The MIC was read as the first well in which growth was reduced to a button <1 mm or replaced by light haze/faint turbidity corresponding to ≥80% reduction compared with the growth control, as defined identically by CLSI and EUCAST (22, 26). Quality control was verified using *E. coli* ATCC 25922 and *P. aeruginosa* ATCC 27853, with MIC values (0.06–0.5 µg/mL) within the acceptable range defined by both standards.

### Breakpoints

Interpretive criteria for FDC were applied according to multiple standards, including those from the CLSI (17), EUCAST (18), and U.S. FDA (19, 27). Both MIC and DD results were evaluated using the respective breakpoints provided by each organization (Table 1).

**TABLE 1:** Interpretive breakpoints for cefiderocol susceptibility testing^*a*^

Organisms	CLSI			EUCAST			U.S. FDA		
	S	I	R	S	R	ATU	S	I	R
Disk diffusion breakpoints (mm)									
Enterobacterales	≥16	9-15	≤8	≥23	<23	21-23	≥16	9-15	≤8
*Pseudomonas aeruginosa*	≥18	13-17	≤12	≥22	<22	20-21	≥22	13-21	≤12
*Acinetobacter baumannii-calcoaceticus* complex	≥15	–	–	<17 will likely be resistant			≥19	12-18	≤11
*Stenotrophomonas maltophilia*	≥15	–	–	<22 will likely be resistant			≥17	–	–
*Burkholderia cepacia*	No breakpoints available								
*Elizabethkingia anophelis*									
MIC breakpoints (μg/mL)									
Enterobacterales	≤4	8	≥16	≤2	>2	–	≤4	8	≥16
*Pseudomonas aeruginosa*	≤4	8	≥16	≤2	>2	–	≤1	2	≥4
*Acinetobacter baumannii-calcoaceticus* complex	≤4	8	≥16	>2 will likely be resistant			≤1	2	≥4
*Stenotrophomonas maltophilia*	≤1	–	–	>2 will likely be resistant			M-100 standard is recognized		
*Burkholderia cepacia*	No breakpoints available								
*Elizabethkingia anophelis*									

^*a*^ ATU, area of technical uncertainty; CLSI, Clinical and Laboratory Standards Institute (17); EUCAST, European Committee on Antimicrobial Susceptibility Testing (18); FDA, Food and Drug Administration (19); I, intermediate; R, resistant; S, susceptible. “–” indicate that no interpretive criteria are defined for that category.

### CA analysis

CA and associated error rates (minor error [mE], major error [ME], and very major error [VME]) were determined by comparing DD interpretations against MIC-based interpretations, which served as the reference standard. An mE was defined as a discrepancy between susceptible (S) and intermediate (I), or between I and resistant (R) interpretations. An ME referred to isolates interpreted as S by MIC but R by the DD method, whereas a VME referred to isolates classified as R by MIC but interpreted as S by the DD method. The CA rate was calculated as the percentage of isolates with identical interpretations between the two methods. For organism–drug combinations where the CLSI guideline does not define an intermediate category for DD method, categorical CA was calculated only for isolates classified as either susceptible or resistant by both methods. Isolates with an intermediate MIC result and no corresponding DD method category were excluded from CA analysis. Error rates were determined by dividing the number of mEs by the total number of isolates, MEs by the number of MIC-S isolates, and VMEs by the number of MIC-R isolates. A minor error was not calculated for EUCAST criteria, as the I category is not defined. CA rates and error rates (mE, ME, and VME) were reported as percentages with 95% confidence intervals (CIs), calculated using the Wilson score method.

## RESULTS

### Susceptibilities to cefiderocol

Susceptibility rates based on DD and BMD methods varied across species and interpretive criteria (Table 2). According to CLSI interpretive criteria, results obtained from disk diffusion and broth microdilution were generally consistent, with high susceptibility observed across most species, except for *E. cloacae* complex, which showed relatively lower rates. The U.S. FDA criteria are identical to those of CLSI for Enterobacterales, resulting in comparable susceptibility rates, but differ for *P. aeruginosa*, *S. maltophilia*, and the *A. baumannii-calcoaceticus* complex. The stricter FDA breakpoints for *A. baumannii-calcoaceticus* complex led to lower susceptibility in this group. In contrast, interpretation based on EUCAST criteria resulted in markedly lower susceptibility rates and reduced agreement between DD and BMD for most species, except for *P. aeruginosa* and *S. maltophilia*, which remained highly susceptible (Table 2).

**TABLE 2:** Susceptibilities of 1,170 isolates of Gram-negative bacilli to cefiderocol using the DD and BMD methods^*a*^

Organism (no. of isolates tested)	Zone diameter (mm)	MIC (μg/mL)			Susceptibility rates (%) by DD/BMD using different interpretive criteria								
					CLSI			EUCAST			U.S. FDA		
	Range	Range	50%	90%	S	I	R	S	R	ATU	S	I	R
*Escherichia coli* (200)	6 to >32	≤0.03 to >32	0.25	4	94.0/92.0	4.5/5.0	1.5/3.0	75.5/87.0	24.5/13.0	10.5/NA	94.0/92.0	4.5/5.0	1.5/3.0
*Klebsiella pneumoniae* (300)	6 to 31	≤0.03 to >32	0.25	4	94.3/93.7	5.3/3.3	0.3/3.0	84.0/88.3	16.0/11.7	11.3/NA	94.3/93.7	5.3/3.3	0.3/3.0
*Klebsiella oxytoca* (40)	12 to 30	≤0.03 to 16	0.06	8	90.0/85.0	10.0/10.0	0.0/5.0	62.5/75.0	37.5/25.0	7.5/NA	90.0/85.0	10.0/10.0	0.0/5.0
*Enterobacter cloacae* complex (100)	12 to 27	≤0.03 to >32	1	8	83.0/84.0	15.0/13.0	2.0/3.0	36.0/71.0	64.0/29.0	26.0/NA	83.0/84.0	15.0/13.0	2.0/3.0
*Pseudomonas aeruginosa* (100)	20 to >32	≤0.03 to 4	0.12	0.25	100.0/100.0	0.0/0.0	0.0/0.0	99.0/99.0	1.0/1.0	1.0/NA	99.0/98.0	1.0/1.0	0.0/1.0
*Acinetobacter baumannii-calcoaceticus* complex (300)	6 to 30	≤0.03 to >32	1	16	89.7/89.7	NA/2.3	10.3/8.0	82.3/88.3	17.7/11.7	NA	64.0/79.3	29.7/9.0	6.3/11.7
*Stenotrophomonas maltophilia* (60)	17 to >32	≤0.03 to 2	≤0.03	0.06	100.0/98.3	NA	0.0/1.7	98.3/100.0	1.7/0.0	NA	100.0/98.3	NA	0.0/1.7
*Burkholderia cepacia* (20)	28 to >32	≤0.03 to 0.06	≤0.03	0.06	NA	NA	NA	NA	NA	NA	NA	NA	NA
*Elizabethkingia anopheles* (50)	13 to 23	0.5 to >16	4	8	NA	NA	NA	NA	NA	NA	NA	NA	NA

^*a*^ ATU, area of technical uncertainty; BMD, broth microdilution; DD, disk diffusion; I, intermediate; NA, not available; R, resistant; S, susceptible.

### Comparison of different criteria for *E. coli*

**Fig 1:**
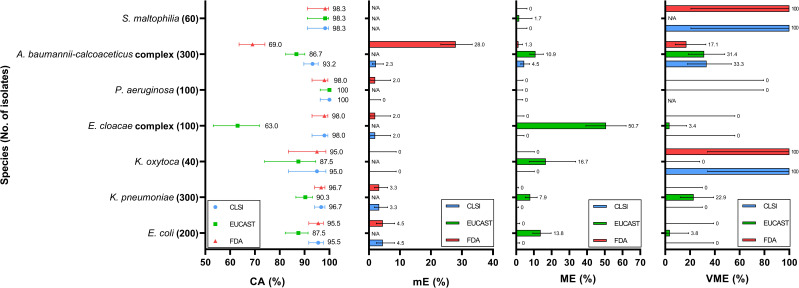
Categorical agreement (CA), minor error (mE), major error (ME), and very major error (VME) rates for each bacterial species. From left to right, the four panels represent CA, mE, ME, and VME, respectively, all aligned to the same *Y*-axis representing bacterial species. Each data point or bar represents the performance of a specific interpretive standard: blue, CLSI; green, EUCAST; and red, U.S. FDA. Error bars indicate 95% confidence intervals.

**Fig 2:**
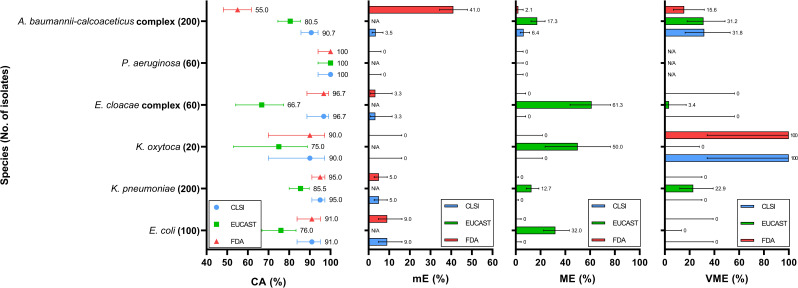
Categorical agreement (CA), minor error (mE), major error (ME), and very major error (VME) rates for carbapenem-resistant isolates among different bacterial species. From left to right, the four panels represent CA, mE, ME, and VME, respectively, all aligned to the same *Y*-axis representing bacterial species. Each data point or bar represents the performance of a specific interpretive standard: blue, CLSI; green, EUCAST; and red, U.S. FDA. Error bars indicate 95% confidence intervals.

**Fig 3:**
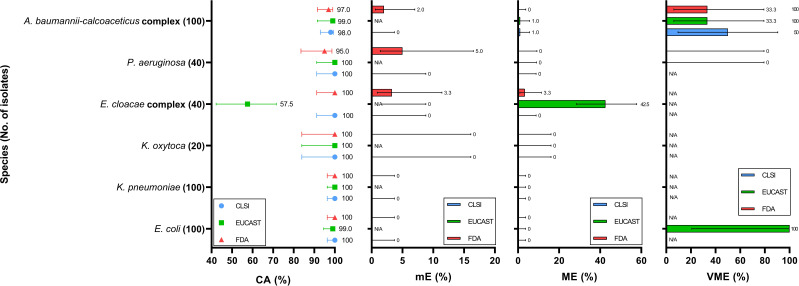
Categorical agreement (CA), minor error (mE), major error (ME), and very major error (VME) rates for carbapenem-susceptible isolates among different bacterial species. From left to right, the four panels represent CA, mE, ME, and VME, respectively, all aligned to the same *Y*-axis representing bacterial species. Each data point or bar represents the performance of a specific interpretive standard: blue, CLSI; green, EUCAST; and red, U.S. FDA. Error bars indicate 95% confidence intervals.

Among the *E. coli* isolates, the CA reached 95.5% (95% CI, 83.8%–95.2%) under the CLSI criteria, with an mE rate of 4.5% (95% CI, 2.4%–8.3%) (Fig. 1; Fig. S1A). No ME or VME was observed. In contrast, EUCAST yielded a lower CA of 87.5% (95% CI, 82.2%–91.4%) and a substantially higher ME rate of 13.8% (95% CI, 9.4%–19.7%), indicating that several isolates interpreted as susceptible by MIC were categorized as resistant by disk diffusion under EUCAST criteria, reflecting a tendency toward false-resistant classification. The U.S. FDA criteria for Enterobacterales are identical to those of CLSI and resulted in the same performance metrics. Under the CLSI criteria, all observed errors were classified as mE. Specifically, three FDC-R isolates were misclassified as FDC-I by the DD method; five FDC-I isolates were misclassified as FDC-S; and one FDC-S isolate was misclassified as FDC-I. In contrast, EUCAST errors predominantly occurred among CR isolates, with 24 FDC-R isolates misclassified as FDC-S by the DD method, accounting for the elevated ME rate (32%) (Fig. 2; Fig. S1B). In addition, the single isolate within the CS group with an MIC of 4  µg/mL (categorized as FDC-R by EUCAST) exhibited a 24 mm inhibition zone and was interpreted as FDC-S by the DD method, resulting in a VME rate of 100% (95% CI, 20.7%–100.0%) (Fig. 3; Fig. S1C).

### Comparison of different criteria for *K. pneumoniae*

For *K. pneumoniae* isolates, the performance under the CLSI criteria was similar to that observed for *E. coli*, with a CA of 96.7% (95% CI, 94.0%–98.2%) and an mE rate of 3.3% (95% CI, 1.8%–6.0%) (Fig. 1; Fig. S2A). No ME or VME was detected. All mEs occurred among CR isolates, including eight FDC-R isolates misclassified as FDC-I and two FDC-I isolates misclassified as FDC-S by the DD method (Fig. S2B and C). Under the EUCAST criteria, the performance was again inferior to that of CLSI, with a CA of 90.3% (95% CI, 86.5%–93.2%), an ME rate of 7.9% (95% CI, 5.2%–11.8%), and a VME rate of 22.9% (95% CI, 12.1%–39.0%). All errors occurred in CR isolates. Specifically, 21 FDC-S isolates were misclassified as FDC-R by the DD method, while 8 FDC-R isolates were misclassified as FDC-S, suggesting that EUCAST breakpoints tend to yield both false-resistant and false-susceptible results among carbapenem-resistant *K. pneumoniae*.

### Comparison of different criteria for *K. oxytoca*

For *K. oxytoca* isolates, the CA was 95.0% (95% CI, 83.5%–98.6%) under the CLSI criteria and 87.5% (95% CI, 73.9%–94.5%) under EUCAST (Fig. 1; Fig. S3A). Among CR isolates, CA was 90.0% (95% CI, 69.9%–97.2%) for CLSI and 75.0% (95% CI, 53.1%–88.8%) for EUCAST (Fig. 2; Fig. S3B). Despite the higher CA under CLSI, both FDC-R isolates were misclassified as FDC-S by the DD method, resulting in a VME rate of 100.0% (95% CI, 34.2%–100.0%). In contrast, the EUCAST criteria produced an ME rate of 50.0% (95% CI, 23.7%–76.3%), with half of the FDC-S isolates misclassified as FDC-R. Both guidelines achieved 100% CA among CS isolates; however, due to the absence of FDC-R isolates in this group, the VME rate could not be determined (Fig. 3; Fig. S3C).

### Comparison of different criteria for *E. cloacae* complex

For *E. cloacae* complex isolates, the CLSI criteria yielded a CA of 98.0% (95% CI, 93.0%–99.4%) (Fig. 1; Fig. S4A). Only two CR isolates were misclassified by the DD method, resulting in an mE rate of 2.0% (95% CI, 0.6%–7.0%), with no ME or VME observed (Fig. 2; Fig. S4B). In contrast, the EUCAST criteria resulted in a substantially lower CA of 63.0% (95% CI, 53.2%–71.8%). The predominant discrepancy was ME, with an overall ME rate of 50.7% (95% CI, 39.3%–62.0%), indicating that the discrepancies were mainly due to false-resistant results. Similar misclassification rates were observed in both CR and CS groups, with ME rates of 61.3% and 42.5%, respectively (Fig. 2; Fig. S4B and C). Additionally, one VME was observed in a CR isolate under the EUCAST criteria.

### Comparison of different criteria for *P. aeruginosa*

For *P. aeruginosa isolates*, all three interpretive criteria demonstrated excellent CA, with both CLSI and EUCAST achieving 100% and U.S. FDA achieving 98% (Fig. 1; Fig. S5A). The only two discrepancies were minor errors observed under the U.S. FDA criteria, both involving CS isolates (Fig. 3; Fig. S5B and C). This may be attributed to the overall MIC distribution being concentrated below 1  µg/mL, with most isolates exhibiting inhibition zones larger than 22 mm.

### Comparison of different criteria for *A. baumannii-calcoaceticus* complex

For *A. baumannii-calcoaceticus* complex isolates, CLSI demonstrated a higher CA rate than EUCAST, with 93.2% (95% CI, 89.7%–95.5%) and 86.7% (95% CI, 82.4%–90.1%), respectively (Fig. 1; Fig. S6A). CLSI exhibited a lower ME rate (4.5% vs 10.9%), while the VME rates between the two guidelines were comparable (33.3% vs 31.4%), suggesting that EUCAST criteria tended to produce more false-resistant results, whereas false-susceptible errors occurred at similar frequencies under both standards. These errors were primarily associated with CR isolates, while both criteria showed similar performance among CS isolates (Fig. 2; Fig. S6B and C). In contrast, the U.S. FDA criteria yielded a notably lower CA of 69.0% (95% CI, 63.6%–74.0%), largely driven by a high mE rate of 41.0% (95% CI, 34.4%–47.9%) in CR isolates (Fig. 1; Fig. S6A and B). Specifically, 56 FDC-S and 16 FDC-R isolates were interpreted as intermediate by the DD method. Additionally, three FDC-I isolates were misclassified as FDC-R, and seven were misclassified as FDC-S.

### Comparison of different criteria for *S. maltophilia*

For *S. maltophilia* isolates, the performance of all three interpretive criteria was nearly identical, with a CA of 98.3% (95% CI, 91.1%–99.7%) (Fig. 1; Fig. S7A). A single isolate accounted for the only observed discrepancy across all criteria. This isolate was classified as FDC-R by MIC according to CLSI and U.S. FDA but was interpreted as FDC-S by the DD method, resulting in a VME under both guidelines. Under EUCAST, the same isolate was interpreted as FDC-S by MIC but FDC-R by the DD method, yielding an ME. Notably, this was the only FDC-R isolate identified under CLSI and U.S. FDA criteria, and no isolate was categorized as FDC-R under EUCAST.

### FDC BMD and DD method results for *B. cepacia* and *E. anophelis*

Currently, no interpretive criteria for cefiderocol susceptibility are available for *B. cepacia* or *E. anophelis* in any of the three major guidelines. Among 20 *B. cepacia* isolates, MICs were uniformly low (≤0.06 µg/mL), whereas 50 *E. anophelis* isolates exhibited higher MICs with MIC_50_ = 4 µg/mL and MIC_90_ = 8 µg/mL. Detailed MIC and inhibition-zone distributions are shown in Fig. S7 and S8.

## DISCUSSION

In this study, across the bacterial species tested, CA values for all interpretive criteria were generally high, exceeding 87.5%, except for EUCAST in *E. cloacae* complex and U.S. FDA in *A. baumannii-calcoaceticus* complex, where lower CA values were observed. Among CR isolates, EUCAST consistently yielded lower CA compared with CLSI, while U.S. FDA showed particularly low CA (63.0%) for *A. baumannii-calcoaceticus* complex. For species without established breakpoints, *B. cepacia* exhibited uniformly low MIC values, whereas *E. anophelis* showed relatively higher MICs.

 Overall, the pattern of categorical discrepancies varied by both species and interpretive standard. Under the CLSI criteria, *A. baumannii-calcoaceticus* complex was more prone to both false-susceptible and false-resistant results.

In contrast, the EUCAST criteria tended to yield false-resistant interpretations for *E. coli* and *E. cloacae* complex, and both false-susceptible and false-resistant results for *K. pneumoniae* and *A. baumannii-calcoaceticus* complex.

The clinical application of cefiderocol primarily lies in the treatment of infections caused by MDR organisms, including CR isolates (28). At the same time, FDC is typically not included in the routine antimicrobial susceptibility testing panels used in clinical microbiology laboratories. As a result, susceptibility testing for FDC is often initiated only after a CR isolate has been identified, rather than as part of the initial testing algorithm. Therefore, the appropriateness and reliability of interpretive criteria become particularly important when applied to CR isolates. We observed that for *E. coli*, *K. pneumoniae*, and *K. oxytoca*, the CLSI criteria yielded 100% CA among CS isolates, with a slight decrease to 90%–95% among CR isolates (Fig. 1 and 2). In contrast, the CA under EUCAST criteria dropped more substantially, from 99%–100% in CS isolates to 75%–85.5% in CR isolates. This discrepancy appears to stem from the distribution of zone diameters relative to MICs. While most CS isolates had MICs of ≤2 µg/mL and inhibition zones of 23 mm or larger, which are classified as FDC-S by EUCAST, CR isolates within the same MIC range were more likely to exhibit inhibition zones smaller than 22 mm, which fall under the EUCAST criterion for FDC-R (Fig. S1 through S3). Notably, among CR isolates of these three species, 15.3% (49/320) fell into this category, representing 20.0% (50/250) of all CR isolates with FDC-S. Even when isolates within the area of technical uncertainty were excluded, 7.2% (23/320) of the CR isolates still resulted in ME, and the ME rate is 9.6% (24/250). These findings suggest that the use of a relatively large zone diameter breakpoint defined by EUCAST may lead to misclassification of FDC-S isolates as resistant when the DD method is used for susceptibility testing.

In this study, EUCAST criteria yielded consistently low CA for *E. cloacae* complex isolates, both in CR and CS groups, with CA rates of 66.7% and 57.5%, respectively, accompanied by high ME rates of 61.3% and 42.5% (Fig. 1). Upon examining the distribution of FDC MICs and corresponding zone diameters, we observed that, compared to other Enterobacterales species in this study, a higher proportion of *E. cloacae* complex isolates with MICs ≤2 µg/mL (FDC-S) exhibited large inhibition zones (>22 mm), which fall under the EUCAST criterion for resistance (Fig. S4). While this pattern was primarily observed in CR isolates of other Enterobacterales, it was also evident among CS isolates of *E. cloacae* complex. In contrast, the CLSI standard, which uses a lower zone diameter breakpoint, did not demonstrate this discrepancy, and both mE and ME rates were substantially lower (2% and 0%, respectively). These findings suggest that when the DD method is used to determine FDC susceptibility for *E. cloacae* complex, EUCAST criteria may lead to higher rates of misclassification regardless of carbapenem resistance status, compared to MIC-based interpretation.

The discrepancies between EUCAST and CLSI criteria can be attributed to both methodological and conceptual differences in how their breakpoints are determined. EUCAST establishes cefiderocol MIC breakpoints primarily on the basis of pharmacokinetic/pharmacodynamic (PK/PD) target attainment and epidemiological cutoff values (ECOFFs). Disk diffusion thresholds are subsequently derived from MIC–zone diameter histograms, a process designed to minimize false-susceptible results by applying more conservative susceptible cutoffs (29). In contrast, CLSI uses an error-rate-bounded approach in which breakpoints are empirically optimized to balance major and very major errors across MIC–zone diameter scatterplots, often incorporating an intermediate category to reduce misclassification (30). These distinct approaches to breakpoint setting, combined with differences in incubation and endpoint reading procedures, likely contributed to the systematic discrepancies in susceptibility interpretation observed between the two systems. Because EUCAST disk diffusion breakpoints are derived from PK/PD and ECOFF analyses rather than direct optimization against MIC categorical agreement, systematic differences between MIC and disk diffusion interpretations are expected. Borderline isolates with MIC values near the susceptible breakpoint may more frequently fall below the EUCAST zone diameter thresholds, resulting in lower categorical agreement and more false-resistant classifications. This feature reflects EUCAST’s conservative design philosophy, which prioritizes avoiding false-susceptible results over maximizing categorical concordance.

We noted that among *A. baumannii-calcoaceticus* complex isolates, the correlation between MIC results and inhibition zone diameters was relatively poor (Fig. 1; Fig. S6). For example, four isolates classified as FDC susceptible based on MIC results (including three FDC-S and one FDC-I according to U.S. FDA criteria) exhibited extremely small inhibition zones (6 mm). This discrepancy may be related to the “trailing growth” phenomenon that has been observed when determining FDC MICs for *A. baumannii* using the BMD method. Trailing growth can complicate MIC interpretation and lead to inaccurate categorization (31). According to CLSI and EUCAST guidelines, MICs should be read at the first well showing either a button of approximately ≤1 mm or a light haze or faint turbidity with a significant (e.g., 80%) reduction in growth compared to the growth control well (17, 22). Although studies have supported the validity of this endpoint determination method as described in the CLSI M100 document, practical interpretation can still be challenging and may contribute to errors in MIC assessment (32).

Although FDC-resistant isolates remain relatively uncommon, increasing reports have documented elevated resistance rates (33–36). The underlying mechanisms of FDC resistance are believed to involve β-lactamases, porin mutations, alterations in siderophore receptors, efflux pumps, and target site modifications involving PBP3, which is the primary target of FDC, particularly in the context of co-expression of multiple β-lactamases (10). For instance, studies have shown that specific KPC mutations can increase FDC MICs in *E. coli*, *K. pneumoniae*, and *E. cloacae* (37), while mutations in AmpC enzymes have also been associated with increased FDC resistance in *E. cloacae* (38). In *A. baumannii*, deletion of iron transport genes such as *piuA*, *pirA*, and *fiuA*, and in *K. pneumoniae*, mutations in *exbD* have been linked to reduced FDC susceptibility (39, 40). In our study, based on CLSI breakpoints, the overall FDC resistance rate was 4.1% (45/1,100), primarily involving *A. baumannii-calcoaceticus* complex (*n* = 24), *K. pneumoniae* (*n* = 9), and *E. coli* (*n* = 6). Among CR isolates, the resistance rate increased to 6.6% (42/640). However, when applying EUCAST criteria, the overall FDC resistance rate tripled to 12.3% (135/1,100) and rose to 20.5% (131/640) among CR isolates. Compared with CLSI, the number of FDC-resistant isolates under EUCAST increased by 9.7-fold in *E. cloacae* complex, 5-fold in *K. oxytoca*, 4.3-fold in *E. coli*, and 3.9-fold in *K. pneumoniae*. These findings highlight how differences in interpretive breakpoints can significantly influence the assessment of resistance rates and complicate the subsequent investigation of resistance mechanisms.

The main limitations of this study include the lack of investigation into other antimicrobial resistance profiles and the underlying mechanisms of carbapenem resistance among the isolates. In addition, the number of FDC-resistant isolates was relatively small, which may have made the VME rate susceptible to fluctuations caused by a few individual cases and resulted in wide 95% CIs*.*

In conclusion, this study demonstrates that when performing FDC susceptibility testing using the DD method, the CLSI interpretive criteria yield results more consistent with MIC-based classifications. In contrast, the EUCAST criteria are more likely to produce discordant results, particularly in CR Enterobacterales and CS *E. cloacae* complex isolates. The U.S. FDA criteria showed greater inconsistency in classifying CR *A. baumannii-calcoaceticus* complex isolates, often resulting in FDC-I interpretations. Additionally, this study provides BMD and DD method data for *B. cepacia* and *E. anophelis*, for which no established breakpoints currently exist.

## Data Availability

The data sets used during the current study are available from the corresponding author on reasonable request.

## SUPPLEMENTAL MATERIAL

Supplemental figures jcm.01255-25-s0001.docx Figures S1 to S8.

ASM does not own the copyrights to Supplemental Material that may be linked to, or accessed through, an article. The authors have granted ASM a non-exclusive, world-wide license to publish the Supplemental Material files. Please contact the corresponding author directly for reuse.
