# Supplementary material for: Interpretive agreement of susceptibility between broth microdilution and disk diffusion methods for cefiderocol, using criteria from the Clinical and Laboratory Standards Institute, European Committee on Antimicrobial Susceptibility Testing, and the Food and Drug Administration
Source: J Clin Microbiol. 2025 Dec 10;64(1):e01255-25. doi: 10.1128/jcm.01255-25 (PMC12802216; doi:10.1128/jcm.01255-25)
Supplement: Supplemental figures — Figures S1 to S8. [file jcm.01255-25-s0001.docx]

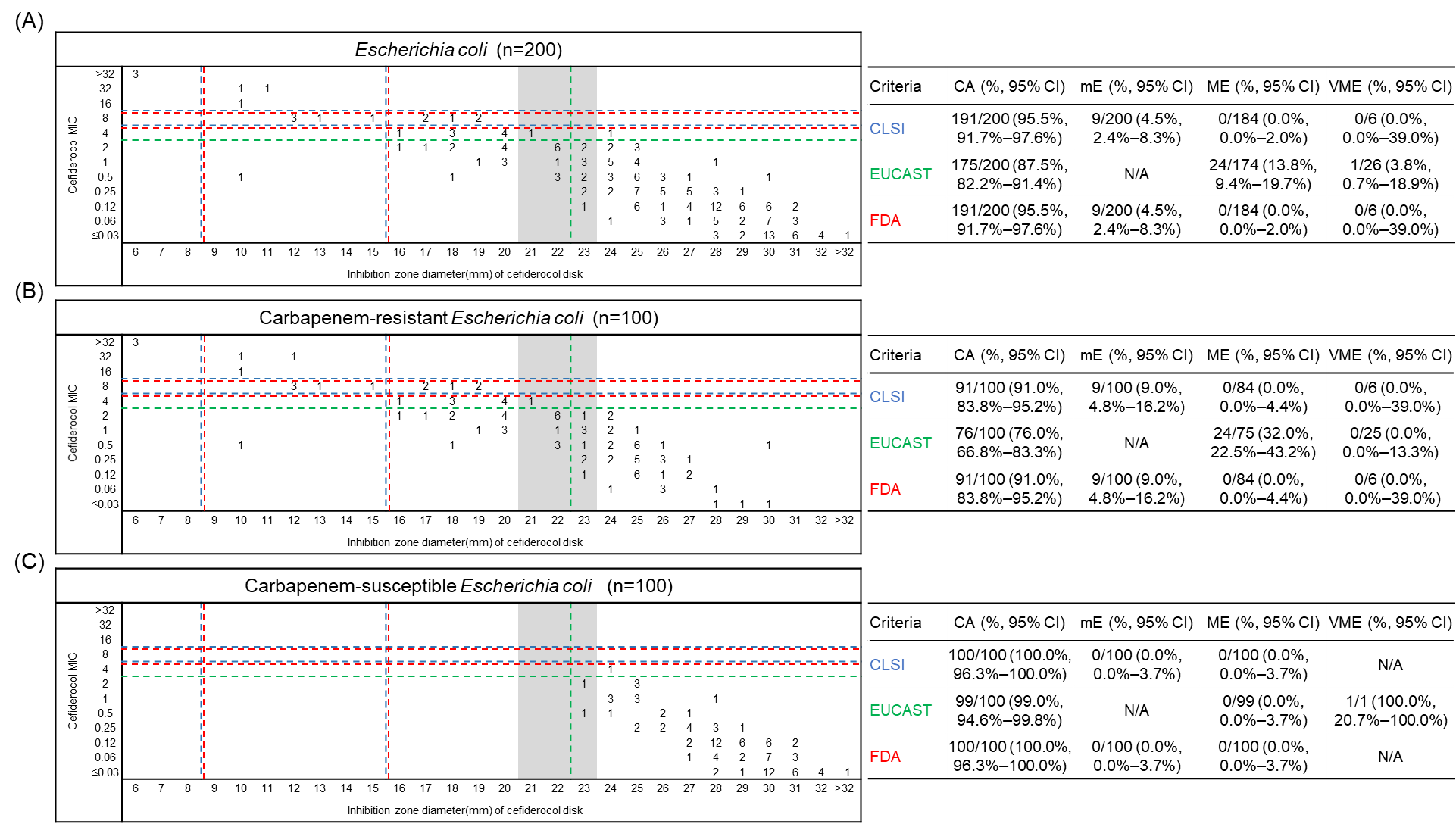


**FIG S1** Distribution of cefiderocol MICs and inhibition zone diameters and corresponding categorical agreement and error rates for *Escherichia coli* isolates. (A) all isolates, (B) carbapenem-resistant (CR) isolates, and (C) carbapenem-susceptible (CS) isolates. In the left panel, horizontal dashed lines indicate MIC breakpoints, and vertical dashed lines indicate disk diffusion zone diameter breakpoints. Breakpoints are colored by guideline: blue for CLSI, green for EUCAST, and red for FDA. Gray shaded areas represent the EUCAST area of technical uncertainty (ATU). The right panels summarize categorical agreement (CA), minor error (mE) rates, major error (ME) rates, and very major error (VME) rates for each guideline.


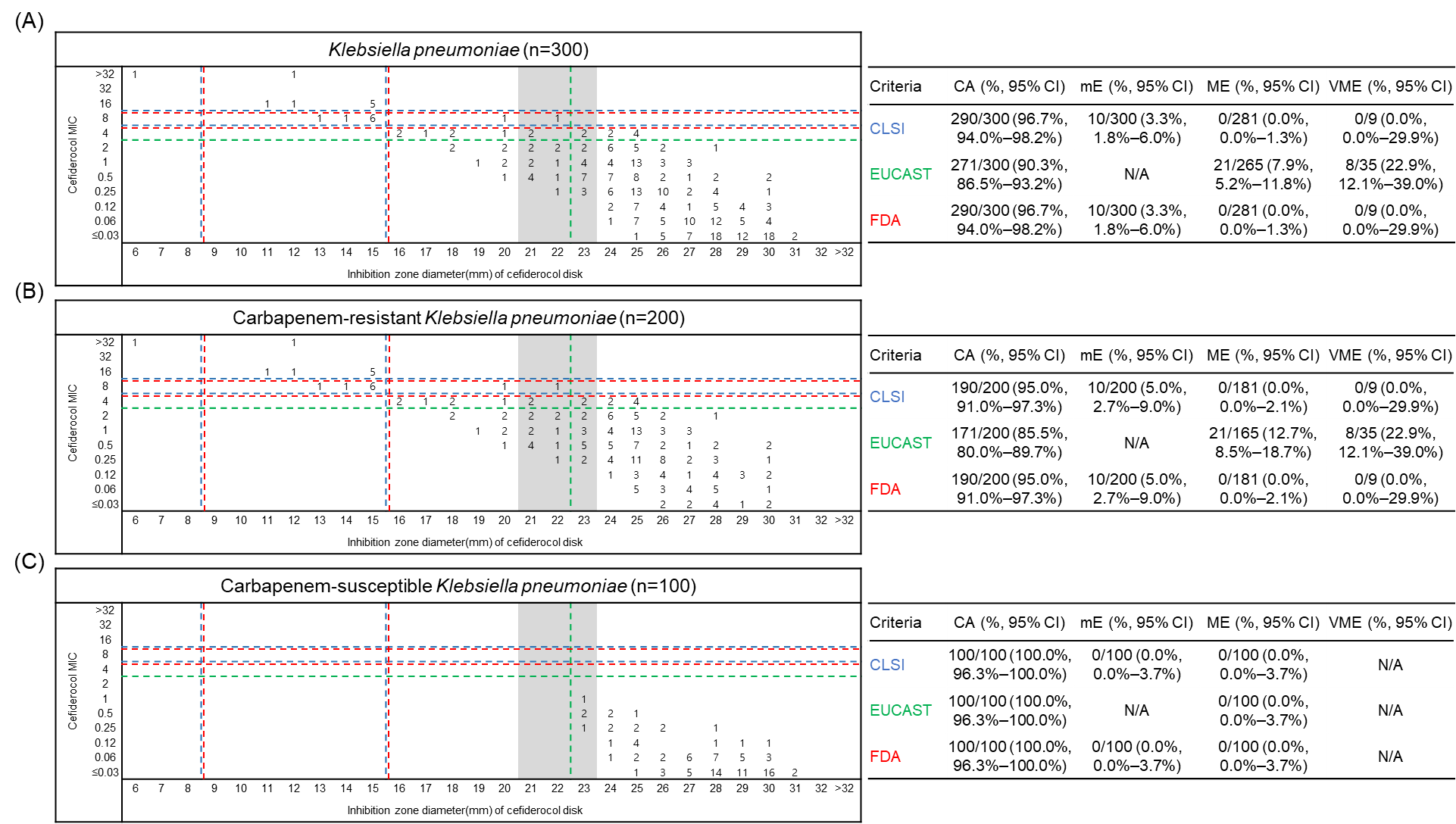


**FIG S2** Distribution of cefiderocol MICs and inhibition zone diameters and corresponding categorical agreement and error rates for *Klebsiella pneumoniae* isolates. (A) all isolates, (B) CR isolates, and (C) CS isolates. In the left panel, horizontal dashed lines indicate MIC breakpoints, and vertical dashed lines indicate disk diffusion zone diameter breakpoints. Breakpoints are colored by guideline: blue for CLSI, green for EUCAST, and red for FDA. Gray shaded areas represent the EUCAST ATU. The right panels summarize CA, mE rates, ME rates, and VME rates for each guideline.


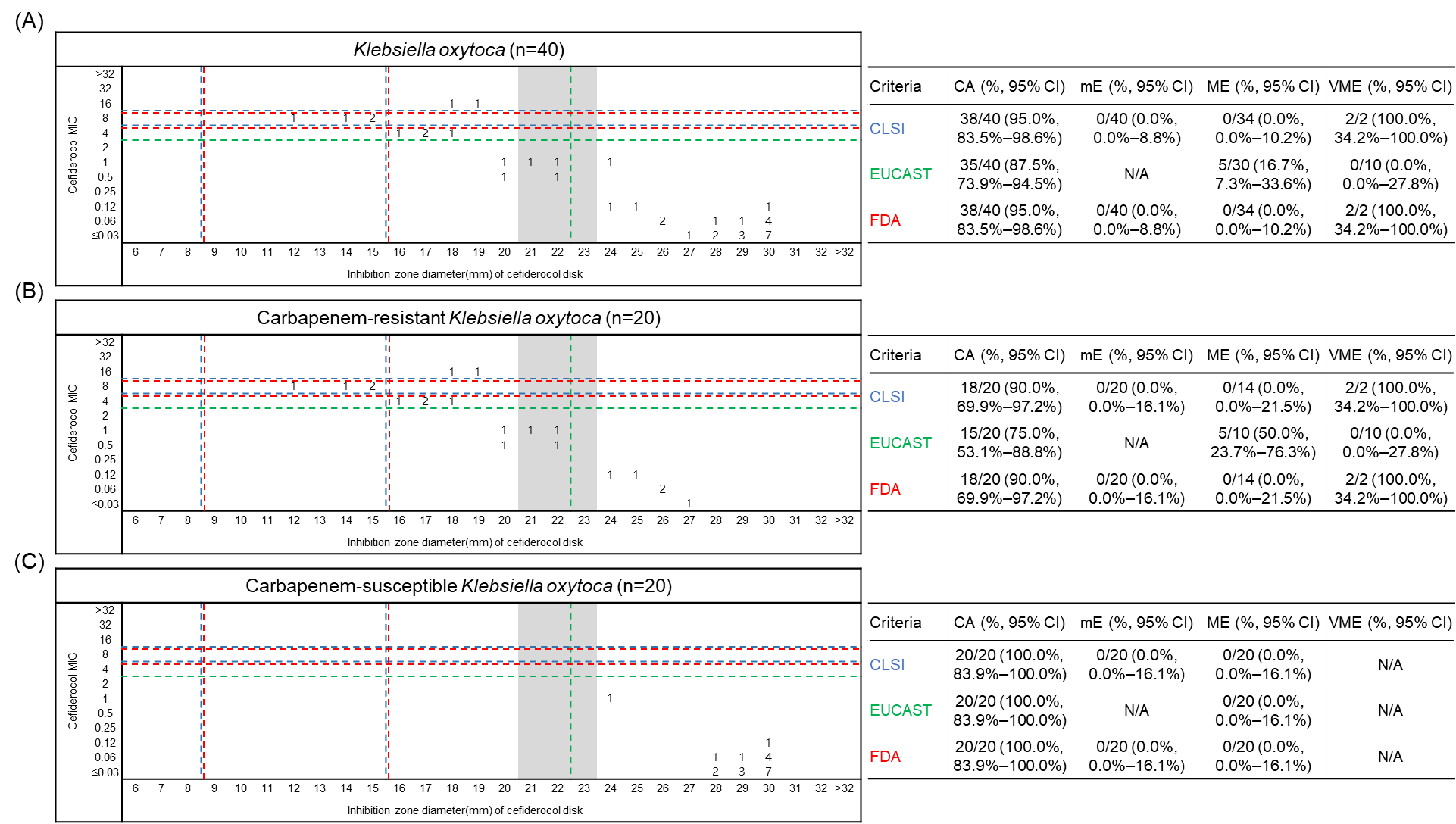


**FIG** **S3** Distribution of cefiderocol MICs and inhibition zone diameters and corresponding categorical agreement and error rates for *Klebsiella oxytoca* isolates. (A) all isolates, (B) CR isolates, and (C) CS isolates. In the left panel, horizontal dashed lines indicate MIC breakpoints, and vertical dashed lines indicate disk diffusion zone diameter breakpoints. Breakpoints are colored by guideline: blue for CLSI, green for EUCAST, and red for FDA. Gray shaded areas represent the EUCAST ATU. The right panels summarize CA, mE rates, ME rates, and VME rates for each guideline.


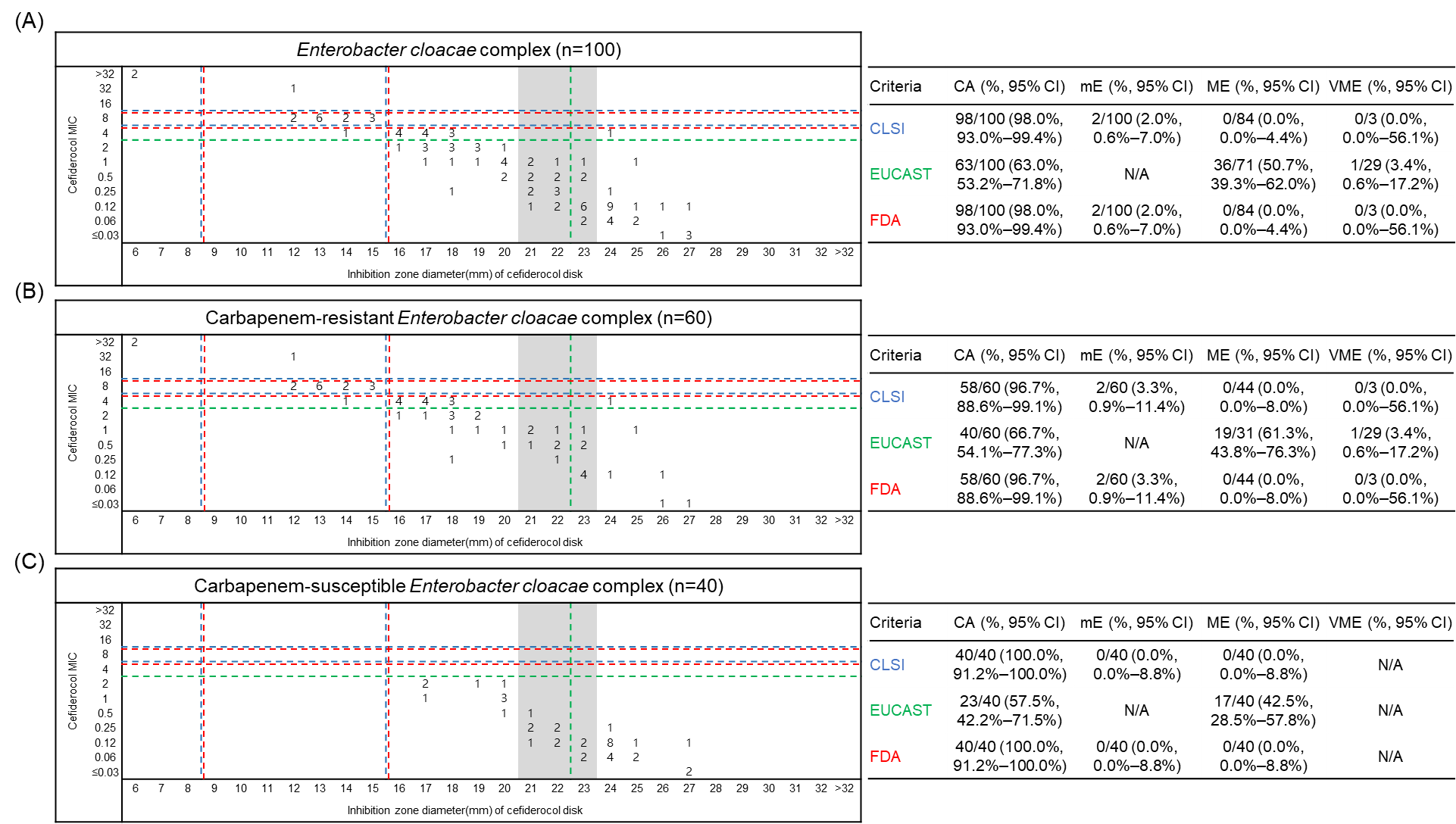


**FIG S4** Distribution of cefiderocol MICs and inhibition zone diameters and corresponding categorical agreement and error rates for *Enterobacter cloacae* complex isolates. (A) all isolates, (B) CR isolates, and (C) CS isolates. In the left panel, horizontal dashed lines indicate MIC breakpoints, and vertical dashed lines indicate disk diffusion zone diameter breakpoints. Breakpoints are colored by guideline: blue for CLSI, green for EUCAST, and red for FDA. Gray shaded areas represent the EUCAST ATU. The right panels summarize CA, mE rates, ME rates, and VME rates for each guideline.


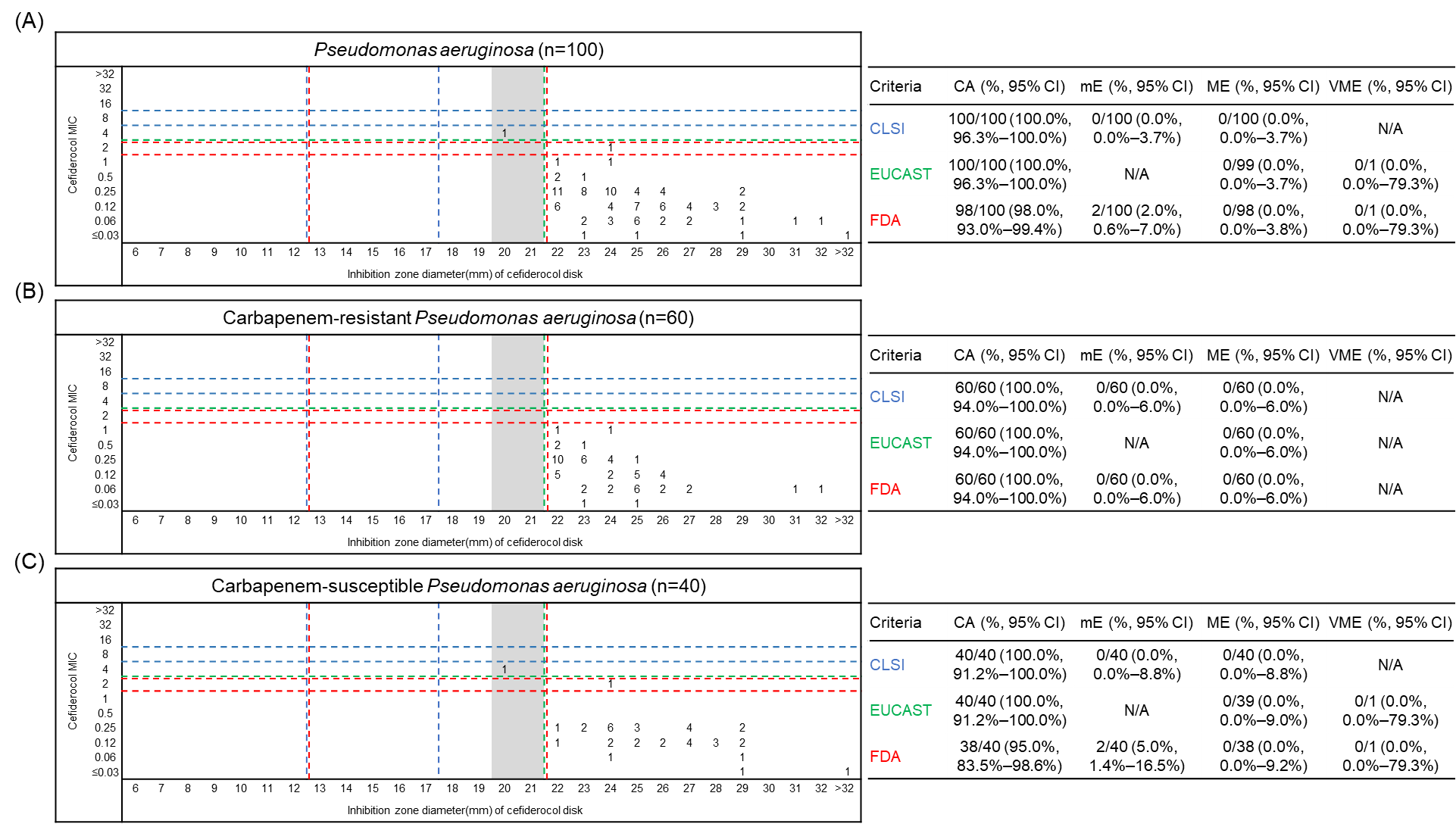


**FIG** **S5** Distribution of cefiderocol MICs and inhibition zone diameters and corresponding categorical agreement and error rates for *Pseudomonas aeruginosa* isolates. (A) all isolates, (B) CR isolates, and (C) CS isolates. In the left panel, horizontal dashed lines indicate MIC breakpoints, and vertical dashed lines indicate disk diffusion zone diameter breakpoints. Breakpoints are colored by guideline: blue for CLSI, green for EUCAST, and red for FDA. Gray shaded areas represent the EUCAST ATU. The right panels summarize CA, mE rates, ME rates, and VME rates for each guideline.


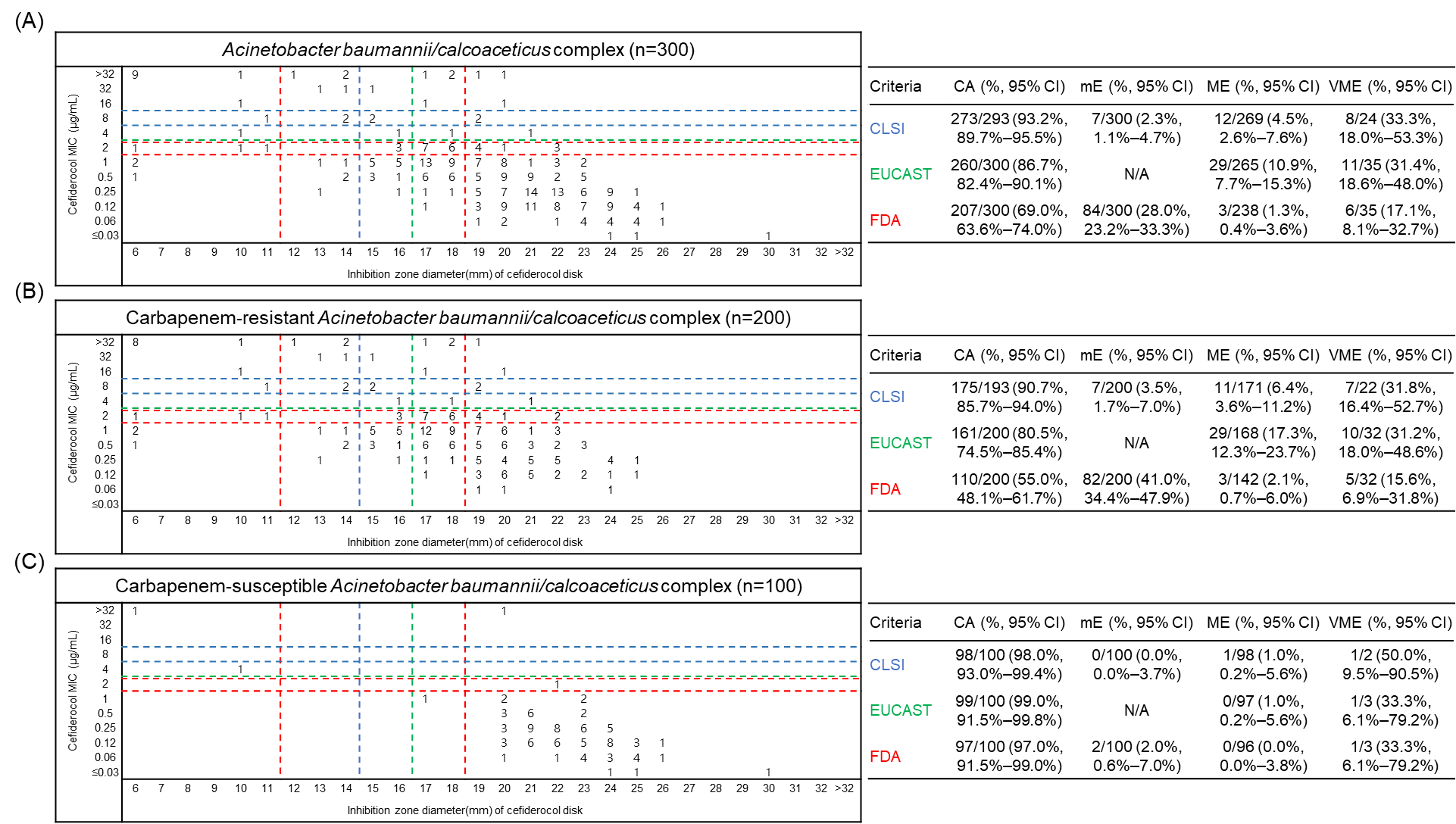


**FIG S6** Distribution of cefiderocol MICs and inhibition zone diameters and corresponding categorical agreement and error rates for *Acinetobacter baumannii/calcoaceticus complex* isolates. (A) all isolates, (B) CR isolates, and (C) CS isolates. In the left panel, horizontal dashed lines indicate MIC breakpoints, and vertical dashed lines indicate disk diffusion zone diameter breakpoints. Breakpoints are colored by guideline: blue for CLSI, green for EUCAST, and red for FDA. The right panels summarize CA, mE rates, ME rates, and VME rates for each guideline.


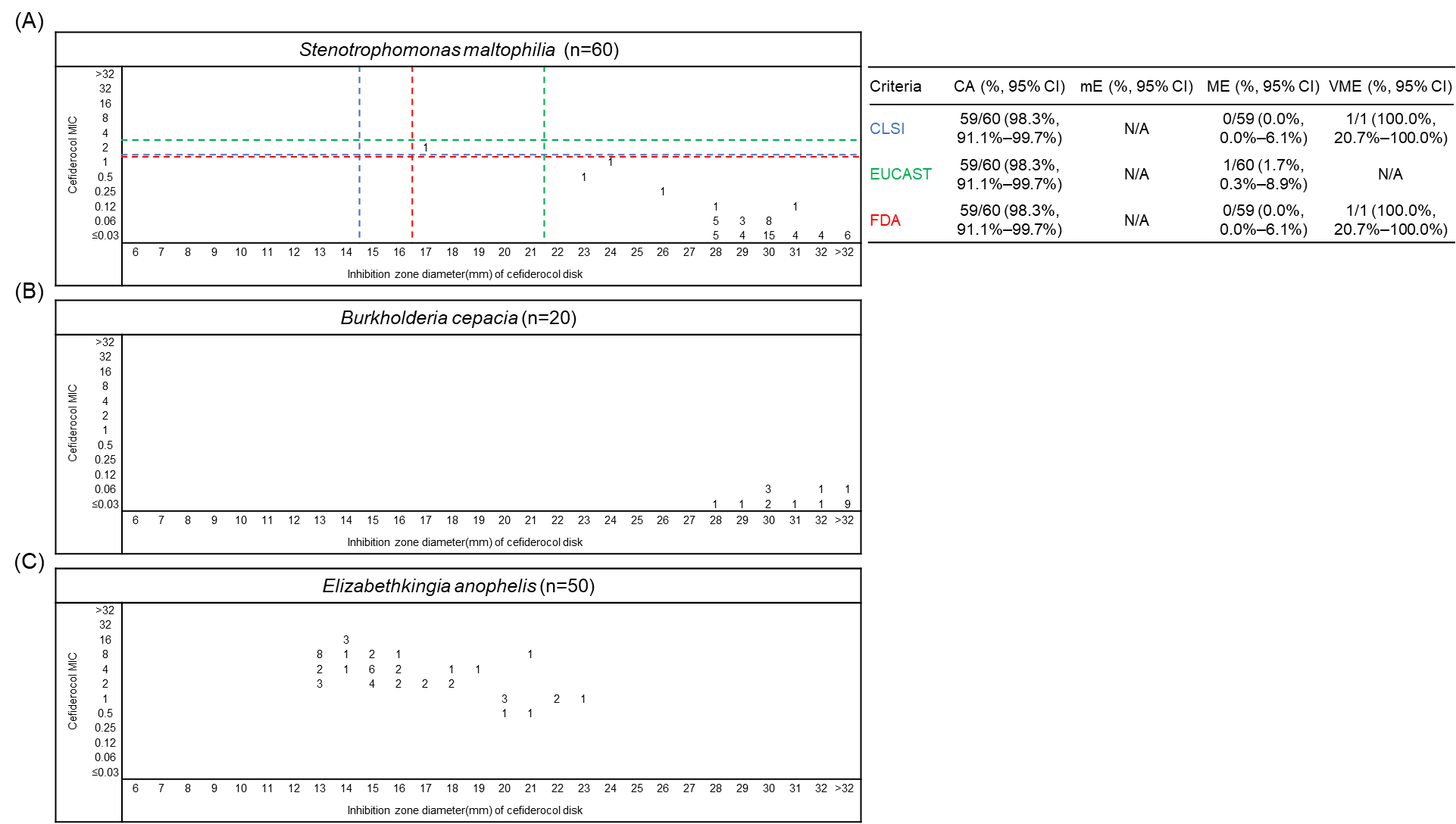


**FIG S7** Distribution of cefiderocol MICs and inhibition zone diameters and corresponding categorical agreement and error rates for *Stenotrophomonas maltophilia*, *Burkholderia cepacia* and *Elizabethkingia anophelis* isolates. (A) *S. maltophilia* isolates, (B) *B. cepacia* isolates, and (C) *E. anophelis* isolates. In the left panel, horizontal dashed lines indicate MIC breakpoints, and vertical dashed lines indicate disk diffusion zone diameter breakpoints. Breakpoints are colored by guideline: blue for CLSI, green for EUCAST, and red for FDA. The right panels summarize CA, mE rates, ME rates, and VME rates for each guideline.

**
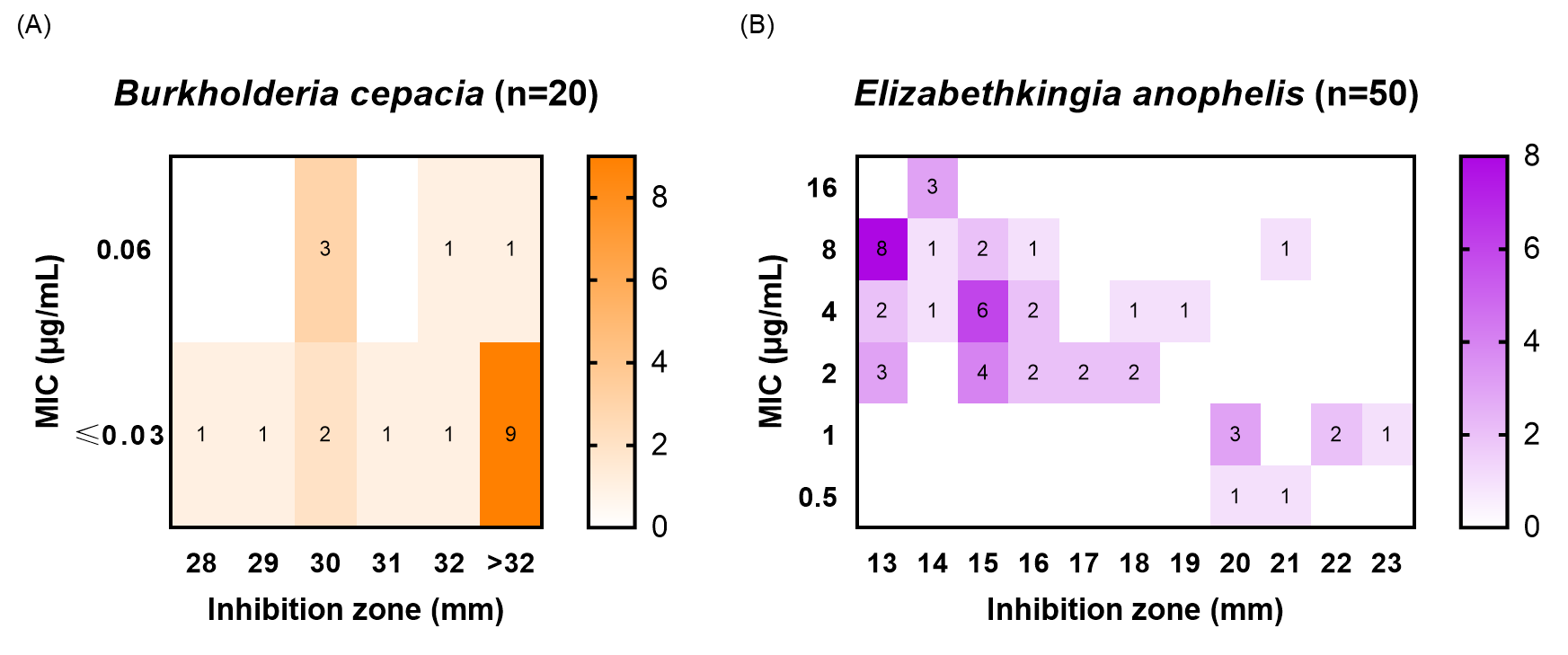
**

**FIG S8** Distribution of cefiderocol minimum inhibitory concentrations (MICs) and inhibition zone diameters in *B. cepacia* and *E. anophelis*. (A) *B. cepacia.* (B) *E. anophelis*. Each cell represents the number of isolates corresponding to a given MIC (µg/mL, y-axis) and inhibition zone diameter (mm, x-axis). Color shading represents the count of isolates per bin; darker shading indicates higher frequency. Zero-count bins are omitted to enhance readability.
